# Oxytocin and Vasopressin Gene Expression in the Brain as Potential Biomarkers for Cannabidiol Therapeutic Efficacy

**DOI:** 10.3390/biomedicines12061273

**Published:** 2024-06-07

**Authors:** Christa M. Frodella, Stephen B. Pruett, Matthew K. Ross, Barbara L. F. Kaplan

**Affiliations:** 1 Department of Comparative Biomedical Sciences, College of Veterinary Medicine, Mississippi State University, Starkville, MS 39762, USA; christa.frodella@gmail.com (C.M.F.); pruett@cvm.msstate.edu (S.B.P.); mross@cvm.msstate.edu (M.K.R.); 2 Center for Environmental Health Sciences, Department of Comparative Biomedical Sciences, College of Veterinary Medicine, Mississippi State University, Starkville, MS 39762, USA

**Keywords:** cannabidiol, cannabinoid, multiple sclerosis, autoimmune, neuroinflammation, oxytocin, vasopressin, gene expression, brain, mice

## Abstract

Over the last several years, there has been increased interest in cannabidiol (CBD) to treat various ailments such as pain, anxiety, insomnia, and inflammation. The potential for CBD as an anti-inflammatory therapy has come, in part, from its demonstrated ability to suppress neuroinflammation in autoimmune diseases, such as the mouse model of multiple sclerosis, experimental autoimmune encephalomyelitis (EAE). The increased use of CBD strongly suggests that more research is necessary to elucidate its safety and efficacy and determine the mechanisms by which it acts. Thus, we conducted two separate studies. In the first, RNA sequencing (RNA-Seq) analysis of brains of female mice undergoing EAE in the presence and absence of CBD was conducted to identify potential genes that mediated its neuroprotective effects when efficacious. In the second, we assessed some of the same genes in male and female mice treated with CBD in the absence of an immune stimulus. Together, these data showed that CBD modestly increased oxytocin (*Oxt*) and arginine vasopressin (vasopressin, *Avp*) gene expression in the brains of mice, regardless of whether there was active inflammation. Overall, these data suggest that *Oxt* and *Avp* might act as biomarkers for CBD exposure.

## 1. Introduction

The dramatic and positive increase in the public perception of cannabidiol (CBD) has not only increased open market availability but has created a pressing need to further research its safety, efficacy, and mechanism(s) of action [1]. At present, purified CBD is only approved as an anticonvulsant under the tradename, Epidiolex^®^ [2]; however, it has been reported to provide a benefit for various conditions, including insomnia, anxiety, substance-use disorders, pain, and inflammation [3,4]. Despite the claims of CBD’s benefit for myriad conditions, its efficacy for these conditions is not yet clear.

A subset of avid users take CBD-enriched extracts or preparations to prevent inflammation [1], and therefore it is necessary to understand CBD’s effects and mechanisms under inflammatory and healthy conditions. We and others have demonstrated that CBD exhibits immunosuppressive efficacy in vivo and in vitro [5]. As one example, CBD was shown to attenuate clinical disease and inflammation in experimental autoimmune encephalomyelitis (EAE), a model to study multiple sclerosis (MS) [6,7,8,9,10,11,12]. These previous studies have shown that the mechanisms by which CBD suppresses EAE involve the induction of suppressor cells [6,7], inhibition of neuronal apoptosis [8], and promotion of neuronal survival [9], all of which prevent neurodegeneration, the suppression of immune cell infiltration into the brain [10,12], and the inhibition of brain cytokines and chemokines possibly via the adenosine A_2A_ receptor [11]. Thus, these data provide robust evidence that CBD attenuates EAE disease through a variety of mechanisms. However, CBD is not always 100% efficacious (i.e., EAE disease was not reduced to 0% incidence in many of the studies [6,7,8,9,10], including ours [12]; although not all studies provided quantitative clinical disease scores [11]). Thus, there is a continued need to elucidate the efficacy and mechanisms by which CBD exhibits anti-inflammatory properties. Of course, this is dependent on the dose, route of administration, and timing of CBD treatment relative to disease. It might also depend on the EAE model system.

The EAE model, while broadly used, can vary widely [13]. One of the most common EAE approaches utilizes active immunization with myelin oligodendrocyte glycoprotein (MOG) emulsified in Complete Freund’s Adjuvant (CFA) followed by two intraperitoneal injections of pertussis toxin (PTx). We purposefully omitted the PTx since it is known to broadly inactivate G protein-coupled receptors (GPCRs) [14], and the receptor through which CBD acts in EAE has not been definitively identified, thereby avoiding inhibition of the putative CBD receptor. PTx omission produces a milder disease with a longer onset and variability in disease incidence, which more closely mimics human MS. Indeed, a recent study in our laboratory showed that the EAE brain transcriptome is comparable to that of active MS lesions in the human brain, underscoring EAE without PTx as a relevant model for MS [15].

Thus, the objectives for this study were initially two-fold: (1) examine the brain transcriptome in EAE without PTx in the presence and absence of CBD treatment; and (2) specifically compare brain transcriptomes in EAE without PTx mice in which CBD produced an attenuation of disease compared to those in which CBD was not effective in reducing disease. To accomplish this, we treated animals with oral CBD (75 mg/kg) for 5 days following disease initiation. We then performed RNA-Seq analysis on brains to identify potential genes that mediated CBD’s neuroprotective effects. Based on the results of the brain transcriptome analysis, we added a third objective in which we examined selected gene targets in the brains of healthy mice treated with CBD to determine if gene targets were modulated similarly by CBD in the absence of inflammation.

## 2. Materials and Methods

These data are a continuation of previously published data characterizing the EAE disease incidence [15]. For the RNA-Seq study, we selected brains from female EAE mice treated with a corn oil (CO) vehicle or CBD to make several comparisons: EAE mice exhibiting clinical signs (EAE/CO symptomatic), EAE mice not exhibiting clinical signs (EAE/CO asymptomatic), EAE mice in which CBD was efficacious (EAE/CBD asymptomatic), EAE mice in which CBD was not efficacious (EAE/CBD symptomatic). It should be noted that these a priori assignments of mice were based on a clinical scoring system commonly utilized in active EAE (in which PTx was used) and might not best reflect our mild disease. Regardless, mice were defined as asymptomatic if they were assigned a clinical sign of 0, which means they did not exhibit any clinical signs starting with the initial clinical sign of tail weakness. A follow-up study using similar CBD dosing as in the EAE study was conducted in male and female mice to confirm selected CBD-induced gene expression changes in the absence of inflammation.

### 2.1. Reagents

CBD was provided by the National Institute on Drug Abuse (NIDA/NIH). The MOG_35–55_ peptide (MEVGWYRSPFSRVVHLYRNGK) was obtained from Biosynthesis (Lewisville, TX, USA). Heat-killed Mycobacterium tuberculosis H37Ra (HKMT) was purchased from Difco/BD Biosciences (Detroit, MI, USA). Complete Freund’s Adjuvant was obtained from Sigma (St. Louis, MO, USA). The following primers were purchased from ThermoFisher (ThermoFisher, Waltham, MA, USA). for quantitative real-time reverse transcription polymerase chain reaction (qRT-PCR): *Tnfa* (Mm00443258_m1), *Ifng* (Mm01168134_m1), *Oxt* (Mm01329577_g1), and *Avp* (Mm00437761_g1).

### 2.2. Animals

Female or male C57BL/6J mice aged 8–12 weeks were purchased either from Envigo (Indianapolis, IN, USA) or Jackson (Bar Harbor, ME, USA). All procedures were approved by the Mississippi State University Institutional Animal Care and Use Committee (protocol number 19-273 to BLFK) in an AAALAC-approved facility. Animals were housed at up to five per cage with unlimited access to food and water. Cages were maintained in a room with a temperature of 22 ± 1 °C, humidity at 40–60%, and 12 h light cycles. As disease progressed, food and water access were ensured by placing food pellets on floor and using longer sipper tubes.

### 2.3. Induction and Assessment of EAE

EAE without PTx was induced similarly to our previous study [12]. Mice were anesthetized using 3% isoflurane and EAE was induced by subcutaneously injecting the MOG_35–55_ peptide emulsion. The emulsion was prepared with vigorous mixing for 30 min of a solution containing 100 µg MOG_35–55_ peptide, 500 µg HKMT, and CFA. Each mouse received 100 µL emulsion delivered at 4 sites (2 at the hips, 2 at the shoulders; 25 µL per site). Control mice received 25 µL saline (SAL) at each site. Twenty-four hours after EAE induction, mice were dosed orally with 75 mg/kg CBD in 100 µL of corn oil (CO) or 100 µL CO for five days. Mice were observed over an 18-day period, and scores were given based on the following scale: 0—Asymptomatic; 0.5—Flaccid tail; 1—Hindlimb paresis/waddling; 1.5—Waddling gait; 2—Unable to prevent being placed in dorsal recumbency; 2.5—Hindlimb dragging; 3—Single hindlimb paralysis; 3.5—Single hindlimb paralysis with other hindlimb dragging; and 4—Complete hindlimb paralysis. Any score above 0 was considered symptomatic. Mice were never allowed to progress past a score of 4 for animal welfare purposes. Scores and samples analyzed were from two, independent cohorts. Mice (*n* = 5 each cohort, total 10 mice each group) were divided into three treatment groups: SAL/CO (control), EAE/CO, and EAE/CBD. SAL/CO mice did not show any clinical signs and were not included in any downstream analyses. A subset of *n* = 6 mice from EAE/CO and EAE/CBD were selected for RNA-Seq analysis. For all subsequent results and discussion of our findings, “EAE” will refer to active immunization with MOG in CFA supplemented with HKMT (and no administration of PTx) unless noted otherwise.

### 2.4. EAE Brain Transcriptomic Analysis

Brains were flash frozen in liquid nitrogen and stored in RNALater at −80 °C on Day 18. RNA isolation, RNA quality evaluation, cDNA library construction, and Illumina RNA sequencing (RNA-Seq) were performed by NovoGene (Sacramento, CA, USA). Reads were paired-end and 150 bp at a depth of 40 million reads. Reads were assessed for quality, trimmed, mapped to the GRCm39 genome (NCBI), and underwent differential expression analysis in the CLC Genomics Workbench (Qiagen, Germantown, MD, USA). Differentially expressed genes (DEGs) with a false discovery rate (FDR) ≤ 0.05 were considered significant.

### 2.5. Pathway Analysis

DEGs from the following comparisons were analyzed with Ingenuity Pathway Analysis (IPA, Qiagen): (1) EAE/CBD symptomatic versus EAE/CO symptomatic; (2) EAE/CBD asymptomatic versus EAE/CO asymptomatic; (3) EAE/CBD asymptomatic versus EAE/CBD symptomatic; and (4) EAE/CO asymptomatic versus EAE/CO symptomatic. Canonical pathways were considered significant with a −log(*p*-value) ≥ 1.3 (equivalent to *p*-value ≤ 0.05) and a |z-score| ≥ 2. Positive and negative z-scores reflected the activation and inhibition of pathways, respectively.

### 2.6. CBD Treatment in Healthy Mice

The purpose of this experiment was to assess whether CBD would increase the gene expression of selected genes in the brain in the absence of inflammation. Male and female mice (*n* = 5) were divided into three groups: sesame oil (SO)/Water, CBD/Water, and CBD only. We tested whether creating an emulsion of CBD oil in water in the gut would affect bioavailability [16,17], thereby potentially further increasing the responsiveness of genes. Mice were dosed with 75 mg/kg CBD or SO via oral gavage for five days. One hour after administration, the water groups received 3 mL/kg water via oral gavage. Mice were euthanized 24 h after the last dose on Day 6. The left side of the brain was flash frozen and submerged in RNALater at −80 °C until being processed for gene expression.

### 2.7. qRT-PCR

Select genes were assessed by qRT-PCR. Total RNA was isolated using the RNEasy Kit (Qiagen), then reversed transcribed with random primers using the High-Capacity cDNA Reverse Transcription Kit (Applied Biosystems/ThermoFisher, Waltham, MA, USA). cDNA was amplified with Taqman primers and probe sets. The 18S gene was used as the housekeeping gene and was amplified in the same reaction as the target genes. Genes were quantified using a Real-Time PCR System Agilent Stratagene Mx3005P (Agilent/ThermoFisher, Waltham, MA, USA). Fold-change was calculated using the ΔΔCt method [18].

### 2.8. CBD Analysis

CBD levels in plasma were determined by LC-MS/MS analysis, as previously described [19]. In brief, plasma proteins were precipitated by the addition of five volumes of ice-cold 1:1 *v*/*v* methanol/acetonitrile containing 20 nM CBD-d9 internal standard. After centrifugation, the supernatant was evaporated to dryness under nitrogen and the residues reconstituted in 100 uL of acetonitrile for LC-MS/MS analysis using the conditions reported [19].

### 2.9. Statistical Analysis

Statistical analyses were performed using GraphPad Prism version 7 (San Diego, CA, USA). The mean ± standard error mean (SEM) was determined for fold-changes for each gene analyzed, and RT-qPCR fold-changes were transformed using natural log(fold-change + 1). A two-way ANOVA was performed, and a *p*-value < 0.05 was deemed significant.

## 3. Results

### 3.1. CBD Effects on EAE

We conducted two separate cohorts of mice with EAE in the presence and absence of CBD using 10 mice per treatment group (Table 1). We used Day 18 as our terminal day based on our previous study [12]. We observed a 50% disease incidence in EAE/CO mice with 40% of EAE/CBD mice still exhibiting clinical signs. This was lower as compared to our previous study in which oral CBD attenuated EAE with a disease incidence of 82% for EAE/CO and 64% for EAE/CBD [12]. This demonstrates that the disease incidence in our mild model was variable but that oral CBD efficacy was also variable. Thus, for this reason, we used differential gene expression (DEG) and pathway analysis to gain insights into the mechanisms of our mild disease incidence and variability in CBD efficacy.

### 3.2. Key DEGs Related to CBD Treatment

To assess the impact of CBD in asymptomatic and symptomatic mice, four comparisons were analyzed from which key DEGs were identified: (1) EAE/CBD symptomatic versus EAE/CO symptomatic; (2) EAE/CBD asymptomatic versus EAE/CO asymptomatic; (3) EAE/CBD asymptomatic versus EAE/CBD symptomatic; and (4) EAE/CO asymptomatic versus EAE/CO symptomatic. The EAE symptomatic in EAE/CBD versus EAE/CO comparison (Table 2) presented 14 DEGs, with 13 that were increased and 1 that was decreased. It was interesting to note that many of the genes increased here were homeobox genes, which are a family of master transcriptional regulators that play key roles in development [20]. The EAE asymptomatic in EAE/CBD versus EAE/CO comparison (Table 3) presented five increased DEGs. Of note here were the genes encoding oxytocin (*Oxt*) and vasopressin (*Avp*) being highly upregulated in CBD-treated brains from mice that were not exhibiting clinical symptoms.

The DEGs for EAE/CBD asymptomatic vs. EAE/CBD symptomatic (Table 4) were analyzed. Again, two genes that were profoundly increased and highly significant were *Oxt* and *Avp* in EAE/CBD asymptomatic versus EAE/CBD symptomatic, similar to EAE/CBD asymptomatic versus EAE/CO asymptomatic (Table 3). The comparison between EAE/CO asymptomatic vs. EAE/CO symptomatic (Table 5) is representative of the mice that were induced with EAE but did not present clinical signs. This comparison reveals the downregulation in asymptomatic mice of several genes associated with inflammation and immunity, including chemokines (*Cxcl9*, *Cxcl10*, and *Ccl5*), genes that encode proteins that are induced by, or interact with, interferons (*Gbp2*, *Gm12250*, *Igtp,* and *Cxcl10*), and major histocompatibility complex (MHC) genes (*H2-Eb1*, *H2-Q6*, and *H2-Q9).* Although the genes *Oxt* and *Avp* were not present in the top 10 DEGs in the EAE/CO asymptomatic versus EAE/CO symptomatic, they were still significantly upregulated, but to a lesser degree. The expression of *Oxt* had a fold-change of 74.01 that was significant (FDR = 3.58 × 10^−4^). Additionally, the expression of *Avp* was also increased (fold-change = 247.33) which was significant (FDR = 2.88 × 10^−9^).

### 3.3. Oxytocin in Brain Signaling Pathway

**Figure 1:**
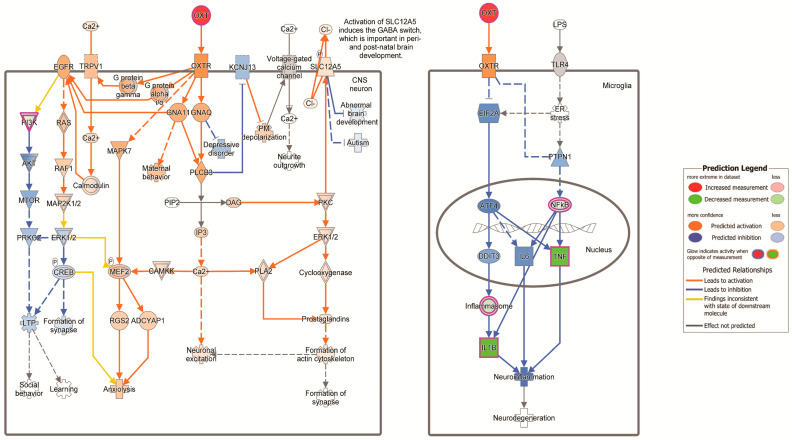
Oxytocin signaling brain pathway. The signaling pathway for the comparison, EAE/CBD asymptomatic vs. EAE/CBD symptomatic, was generated from IPA. Oxytocin was significantly increased (red) in CBD-treated asymptomatic mice, which is predicted to lead to downstream decreases (green) in tumor necrosis factor (TNF), interleukin-1B (IL1B), inflammasome, and PI3K. CO, corn oil (vehicle).

Since the induction of the *Oxt* and *Avp* genes was significantly increased by CBD in mice that did not exhibit clinical symptoms, we investigated the oxytocin pathway. In IPA, the “Oxytocin in Brain Signaling Pathway” was significant in the comparison between EAE/CBD asymptomatic versus EAE/CBD symptomatic. This pathway was activated (z-score = 2.121) and significant at a *p*-value = 0.0378. In contrast, the comparison between EAE/CO asymptomatic versus EAE/CO symptomatic presented an insignificant (*p*-value = 0.0687) but activated (z-score = 2.449) oxytocin pathway. As noted in the pathway, oxytocin’s engagement of its receptor can occur in either neurons or microglial cells and in microglial cells would lead to the inhibition of several pro-inflammatory cytokines (Figure 1).

### 3.4. qRT-PCR Validation of Gene Expression

**Figure 2:**
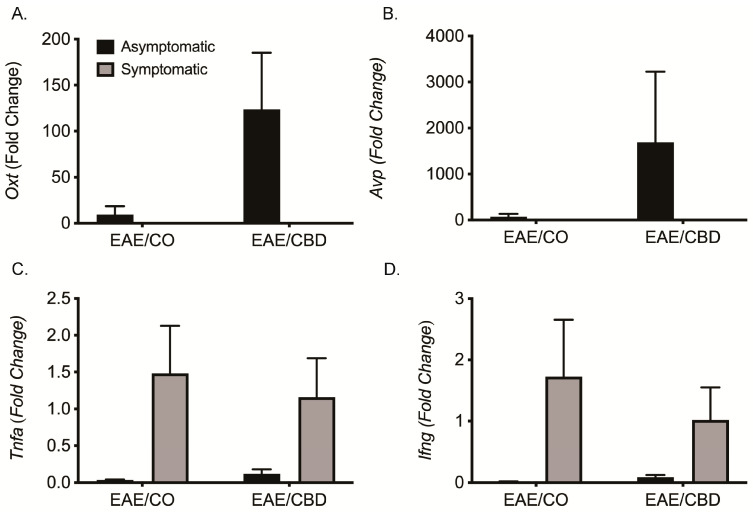
Gene expression confirmation using qRT-PCR. Female EAE mice were treated with CO or CBD for 5 days after disease initiation. Brains were harvested on Day 18. Samples used for RNA-Seq analysis were also used to confirm gene expression by qRT-PCR. (**A**) *Oxt*; (**B**) *Avp*; (**C**) *Tnfa*; and (**D**) *Ifng*. Bars are the mean ± SEM. Asymptomatic EAE values were used as the fold-change analysis comparators. None were statistically significant. CO, corn oil (vehicle).

To validate the expression of *Oxt* and *Avp*, qRT-PCR was performed in the brains used for RNA-Seq analysis. The expression levels are compared to EAE/CO asymptomatic for all genes. Although the expression levels for *Oxt* and *Avp* were not statistically significant, the EAE/CBD asymptomatic groups showed an increased trend in both genes as compared to all other groups (Figure 2A,B). To address the possible anti-inflammatory effects of CBD in EAE, the gene expression for *Tnfa* and *Ifng* was also conducted and again, while there were no statistically significant differences, the trend shows a relatively lower expression for both genes in mice treated with CBD that exhibited no symptoms (Figure 2C,D). These results suggest that the upregulation of *Oxt* and *Avp* might better serve as biomarkers of CBD exposure than the downregulation of pro-inflammatory mediators.

### 3.5. CBD Effects in Brains of Healthy Mice

**Figure 3:**
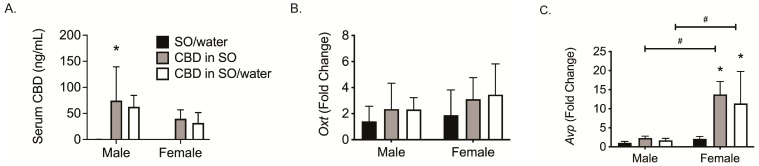
Gene expression assessment in naïve mice using qRT-PCR. Untreated (healthy) male and female mice (*n* = 5) were treated with SO/water, CBD in SO, or CBD in SO/water for 5 days. One hour after the last dose, blood and brains were collected. (**A**) Serum CBD was quantified by LC-MS/MS; (**B**,**C**) *Oxt* and *Avp* gene expression in the brain. Bars are the mean ± SEM. SO/water values were used at the fold-change analysis comparators. * *p* < 0.05 as compared to SO/water mice; # *p* < 0.05 sex differences between same treatment groups. SO, sesame oil (vehicle).

Next, we examined whether *Oxt* and *Avp* gene expression would be increased by CBD in the absence of inflammation or immune stimulation. Male and female mice received the same dosage of CBD as in the EAE study (i.e., 75 mg/kg daily for 5 days via oral gavage). We quantified the serum CBD levels at 24 h after the last dose and found an average of ~40–80 ng/mL regardless of whether we added water in an attempt to increase the serum concentrations as reported (Figure 3A) [17]. In the brains of healthy mice, *Oxt* gene expression was slightly elevated with CBD treatment in both sexes (Figure 3B). *Avp* gene expression was slightly increased in male mice but was significantly upregulated in the brains of female mice by CBD (Figure 3C). These results suggest that the brain expression of *Oxt* or *Avp* might serve as a biomarker of CBD exposure regardless of the presence of inflammation.

## 4. Discussion

The objectives of this work were to identify brain transcriptomic mechanisms that could: (1) identify genes involved with CBD’s ability to attenuate EAE disease; (2) account for the variable efficacy of CBD; and (3) suggest potential biomarkers for CBD exposure. Initially, we intended to simply compare CBD- versus vehicle-treated mice in EAE but took advantage of the variable CBD efficacy to identify differential genes associated with CBD providing protection from disease. While splitting out the groups in this way reduced the number per group (which is a limitation of our study), we were able to gain several insights. We determined that several homeobox genes were upregulated in CBD-treated mice that still showed disease (i.e., when CBD was not efficacious), while *Oxt* and *Avp* were robustly upregulated in CBD-treated mice that did not show disease (i.e., when CBD was efficacious). Finally, we noted that *Oxt* and *Avp* were also modestly upregulated in the brains of CBD-treated healthy mice, suggesting that *Oxt* and *Avp* gene upregulation might serve as biomarkers of CBD exposure.

This work confirmed other studies from our lab showing that active EAE without PTx results in a milder disease with a longer onset [12,15,21,22]. Our reason for omitting PTx is to avoid any inactivation of putative GPCRs through which CBD might be acting, since PTx inactivates Gi/o [14], and the CBD receptor in EAE has not yet been definitively identified. The brains were grouped a priori for RNA-Seq analysis based on the current scoring rubric, from which the minimum score of 0.5 is a loss of tail tone, and sometimes results in no clinical score [12,21,22,23,24,25]. However, we have observed neuroinflammation in our EAE mice even when assigned a clinical score of 0 [26], which has prompted us to consider the possibility of updating our scoring rubric to better reflect our mild disease state. Despite the modest disease as assessed by the current rubric, we know this model is relevant because the transcriptomes of the brains from our EAE mice closely resembled brain transcriptomes from MS individuals in which both active and inactive lesions were found [15].

This study identified that homeobox genes were putatively involved in EAE or were upregulated in mice when CBD was not efficacious in EAE. Homeobox genes encode transcription factors important during development and in adulthood [20]. In humans, homeobox genes, including *HOXA5*, were preferentially expressed in the spinal cord as compared to the brain [27]. *HOXA5* also interacted with two transcription factors, *SMAD1* and *SOX2*, which were previously identified in MS spinal cords as being associated with astrocytosis/gliosis [27]. However, in another study it was noted that HOXB3 protein expression in the cerebral spinal fluid was relatively low in individuals who progressed from an acute episode of neurological disturbance to MS [28]. These studies, together with our results, confirm that homeobox genes are likely involved in EAE and MS, and some might serve as targets of CBD.

The most significant finding in this work was that CBD increased the gene expression of *Oxt* and *Avp* in the brains of mice that were asymptomatic with CBD (i.e., CBD was efficacious in reducing or preventing disease). This was consistent with our modest results in the brains of naïve mice and with another report showing that *Oxt* was increased in the prefrontal cortex of rats treated with CBD [29]. Oxytocin and vasopressin are neuropeptides that are synthesized in the paraventricular and supraoptic nuclei of the hypothalamus and are released from the posterior pituitary into the periphery and act through GPCRs [30]. Thus, a limitation of our study was that we did not determine the effects on *Oxt* and *Avp* in specific brain regions, which is an important next step for our studies.

Although the peptides only differ by two amino acids, their primary roles are distinct. Oxytocin is involved in uterine contractions and milk ejection during labor and breastfeeding. In contrast, vasopressin regulates water retention and blood pressure through its effects on the kidneys [31]. Despite their distinctive roles, both oxytocin and vasopressin have been shown to impact the immune system. Oxytocin secretion is a part of the neuroendocrine-immune network that takes on various roles including the development of the immune system and the inhibition of inflammation [32]. Oxytocin receptors are found in many immune cells, including CD8+ T cells [33] and monocytes [34], and several studies have shown that oxytocin can reduce pro-inflammatory mediators [34,35]. In contrast, vasopressin has been shown to have an opposing role in the immune system, with one study reporting that vasopressin exacerbated inflammation in response to brain trauma [36]. In EAE, blocking vasopressin decreases blood–brain barrier permeability and reduces clinical signs [37]. How CBD-induced increased gene expression for *Oxt* and *Avp* contributes to its anti-inflammatory efficacy is not yet clear, but vasopressin can also attenuate the inflammatory response under certain conditions [38]. At the neuronal level, it has been shown that transient receptor potential V2 (TRPV2), a receptor to which CBD binds [39], co-labels with both oxytocinergic and vasopressinergic neurons [40]. Another potential link was shown between the endocannabinoid, anandamide, and oxytocin. Wei et al. showed that oxytocin drove anandamide signaling at cannabinoid 1 receptors (CB_1_) [41]. Since CBD has also been shown to elevate anandamide levels through the inhibition of fatty acid amide hydrolase, which breaks down anandamide (at least in rodents) [42], it is possible that CBD works with oxytocin to potentiate the endocannabinoid system through CB_1_. As cannabinoids are known to attenuate neuroinflammation via CB_1_ [43] and the loss of CB_1_ exacerbated our EAE model [26], this is a potential mechanism for the association between the neuroprotective effects of CBD and oxytocin.

It has been reported that vasopressin is generally higher in males than females; for instance, it was shown that brain *Avp* gene expression was higher in males across several times during development [44] and that males possess a higher number of vasopressin-immunopositive neurons in certain brain areas [45]. In this study, however, females expressed higher levels of *Avp* gene expression in the brain in response to CBD in the absence of an inflammatory insult. This could represent a sex-specific response to CBD. As noted below, it is not likely to be due to systemic metabolism differences since serum CBD levels were similar, but there have been studies noting differential sensitivity to CBD depending on sex. As one example using a pain model, the sensitivity to CBD changed significantly during the estrous cycle with females in late diestrus exhibiting increased sensitivity to CBD as compared to proestrus [46]. We cannot definitively claim that the stage of the estrous cycle dictated the enhanced female response of *Avp* gene expression to CBD in our study; more studies are needed to examine sex differences in CBD responsiveness and potential efficacy.

As part of our determination that CBD increased *Oxt* and *Avp* expression in the brains of mice exposed in the absence of a neuroinflammatory insult, we determined the serum levels of CBD in mice 24 h after a 5-day oral administration of CBD. Although it has been suggested that dosing with water following oil might increase plasma or serum concentration through the formation of an emulsion in the gut [17], we did not detect a difference in serum CBD. The data confirmed previous studies showing that oral administration of CBD, even over a 5-day period, results in only ng/mL serum levels, suggesting a low bioavailability [47]. The low serum CBD levels after oral dosing might account for some of the variable efficacy of CBD in this study. In a previous study in our laboratory with our mild EAE model, oral CBD did exhibit immunosuppressive efficacy but did not completely resolve inflammation or neuroinflammation [12]. Certainly, this is related to dosage, but 75 mg/kg is already a relatively high oral dose. A comparison of CBD’s efficacy following oral administration as compared to other routes of administration in our EAE model is just one of several future studies that can be considered based on these findings.

There is evidence in humans that CBD exhibits anti-inflammatory effects. While there are limited studies in which only CBD (as compared to a CBD/THC combination treatment) was evaluated in MS [48], other studies demonstrate that CBD possesses anti-inflammatory efficacy in individuals with diagnosed chronic periodontitis [49] or inflammation associated with cocaine-use disorder [50]. Another study showed that the oral administration of CBD reduced LPS-stimulated TNF-α ex vivo [51]. There is one clinical trial in which CBD was evaluated for osteoarthritis knee pain in humans and CBD as a supplemental analgesic was not effective, although there were no inflammation endpoints evaluated [52]. Together, these studies demonstrate that CBD has the potential to exhibit anti-inflammatory efficacy in humans, but that it is variable, similar to our effects in this study. It is likely that CBD’s efficacy depends on the dose, route of administration, duration, presence of other drugs, and degree of immune system activation.

## 5. Conclusions

This study allowed us to decipher part of the mechanism by which CBD protects from neuroinflammation in EAE and also identify transcriptomic reasons to account for CBD’s variable efficacy. A novel feature of this study is that instead of simply averaging the results for each control and treatment group as is typically conducted in experiments like this, we distinguished between mice that had an effective response to CBD treatment and mice that did not. This better reflects the “real world” situation in human patients in which treatment effects can vary substantially between individuals. Most importantly, we showed that in CBD-treated EAE mice without disease, the brain expression of the *Oxt* and *Avp* genes was significantly upregulated. At this time, it is not clear if *Oxt* and *Avp* gene expression is upregulated in neurons, astrocytes, or immune cells (or more than one of these). Moreover, it is not clear in which brain region the upregulation occurs. Although to a lesser extent, CBD also increased the brain gene expression of *Oxt* and significantly increased the brain expression of *Avp*, at least in females, in the absence of an inflammatory stimulus. Thus, these data suggest that the brain expression of *Oxt* and *Avp* might be biomarkers of CBD exposure, regardless of an inflammatory stimulus. Overall, studies such as these that focus on pathways activated in response to an efficacious treatment might suggest novel therapies that more selectively target pathways known to regulate pathological changes in MS, which would perhaps exhibit less overall immunosuppression, which remains one of the most important categories of side effects for drugs that target a component of the immune system.

## Figures and Tables

**Table 1:** Clinical scores on Day 18.

EAE/CO	Score	EAE/CBD	Score
21A	2	**26A**	**0**
**22A**	**0**	**27A**	**0**
**23A**	**0**	**28A**	**3**
**24A**	**2.5**	29A	0
25A	0	**30A**	**3**
**21B**	**2.25**	**26B**	**0**
**22B**	**0**	27B	0
23B	0	28B	2.5
24B	2	29B	0
**25B**	**2.25**	**30B**	**4**

Two separate cohorts (designated A or B) of mice were conducted using 5 mice per group per cohort (total 10 mice per group). Brains from mice with the bold scores were submitted for RNA-Seq. Groups (*n* = 3) were defined as EAE/CO symptomatic (24A, 21B, 25B); EAE/CO asymptomatic (22A, 23A, 22B), EAE/CBD symptomatic (28A, 30A, 30B), and EAE/CBD asymptomatic (26A, 27A, 26B). Mice were considered asymptomatic if they did not exhibit the initial clinical sign of tail weakness. CO, corn oil (vehicle). The EAE/CO subset of these data was previously published [15].

**Table 2:** Symptomatic mice only—EAE/CBD versus EAE/CO ^a^ DEGs (*n* = 3 per group).

Name	Fold Change	FDR
*Hoxb5*	522.45	1.07 × 10^−6^
*Hoxb8*	258.31	1.40 × 10^−5^
*Hoxa5*	307.5	2.07 × 10^−5^
*Dbh*	50.37	5.10 × 10^−5^
*Slc6a2*	249.99	7.63 × 10^−5^
*Hoxb6*	373.91	7.63 × 10^−5^
*Hoxb3*	224.05	2.56 × 10^−4^
*Hoxc4*	104.23	2.49 × 10^−3^
*Hoxa4*	143.68	3.12 × 10^−3^
*Trh*	21.99	3.35 × 10^−3^
*Chat*	33.53	9.85 × 10^−3^
*Pde6a*	−28.4	0.02
*Hoxb2*	45.24	0.04
*Glra1*	45.39	0.05

^a^ CO, corn oil (vehicle).

**Table 3:** Asymptomatic only—EAE/CBD versus EAE/CO ^a^ DEGs (*n* = 3 per group).

Name	Fold Change	FDR
*Oxt*	120.67	3.28 × 10^−5^
*Avp*	38.95	3.12 × 10^−4^
*Capn11*	211.15	3.12 × 10^−4^
*Iqschfp*	105.47	6.10 × 10^−3^
*Pmch*	9.6	0.03

^a^ CO, corn oil (vehicle).

**Table 4:** EAE/CBD asymptomatic versus EAE/CBD symptomatic DEGs (*n* = 3 per group).

Name	Fold Change	FDR
*Oxt*	1311.49	0
*Cxcl9*	−52.68	0
*Cxcl10*	−41.38	0
*Ly6i*	−166.58	0
*Avp*	323.08	3.96 × 10^−13^
*F10*	−203	6.60 × 10^−13^
*Gbp6*	−25.82	1.13 × 10^−12^
*Igtp*	−25.05	1.48 × 10^−12^
*Cfb*	−26.96	4.62 × 10^−12^

**Table 5:** EAE/CO ^a^ asymptomatic versus EAE/CO ^a^ symptomatic DEGs (*n* = 3 per group).

Name	Fold Change	FDR
*Gbp2*	−30.13	0
*Cxcl9*	−689.66	0
*Cxcl10*	−121.38	0
*Gm12250*	−47.14	0
*Igtp*	−30.37	0
*Ccl5*	−258.3	0
*Psmb8*	−20.91	0
*H2-Eb1*	−41.57	0
*H2-Q6*	−42.15	0
*H2-Q9*	−45.82	0

^a^ CO, corn oil (vehicle).

## Data Availability

The mouse brain EAE datasets generated and/or analyzed during the current study are available in the NCBI gene expression omnibus (GEO) GSE262437 (https://www.ncbi.nlm.nih.gov/geo/query/acc.cgi?acc=GSE262437) and were accessed on 24 August 2021 for this manuscript.
